# The Microscopic Anatomy of the Human Body in Health and Disease

**Published:** 1847-10-01

**Authors:** 


THE MICROSCOPIC ANATOMY OF THE HUMAN BODY IN HEALTH AND DISEASE. Illustrated with numerous Drawings in Colours. By *Arthur Hill Hassall.* London : Highley, 1847.

THE Plates of this most useful work which have lately appeared, especially those of Part X., are extremely accurate and well executed. Those of our readers who have examined the figures illustrative of the minute texture and development of bone, will, we are assured, agree in the opinion here expressed. The drawing (Plate 33, fig. 6) taken from a specimen prepared by Mr. Tomes, showing the coloration of the walls of the Haversian Canals from the effect of madder, is particularly interesting as indicating the share taken by the endosteum in the formation of new bone. We can, with much satisfaction, strongly recommend Mr. Hassall's Microscopic Anatomy to all who desire to become acquainted with that most attractive branch of the science of organization, and it is fortunate that the extremely moderate price at which these plates are published, renders them generally attainable. We trust the whole work will be speedily completed, and that the author will receive that general support to which his labours so well entitle him.

